# Speeding through cell cycle roadblocks: Nuclear cyclin D1-dependent kinase and neoplastic transformation

**DOI:** 10.1186/1747-1028-3-12

**Published:** 2008-09-02

**Authors:** Laura L Pontano, J Alan Diehl

**Affiliations:** 1 The Abramson Family Cancer Research Institute, Department of Cancer Biology, University of Pennsylvania, Philadelphia, PA 19104, USA

## Abstract

Mitogenic induction of cyclin D1, the allosteric regulator of CDK4/6, is a key regulatory event contributing to G1 phase progression. Following the G1/S transition, cyclin D1 activation is antagonized by GSK3β-dependent threonine-286 (Thr-286) phosphorylation, triggering nuclear export and subsequent cytoplasmic degradation mediated by the SCF^Fbx4-αBcrystallin ^E3 ubiquitin ligase. Although cyclin D1 overexpression occurs in numerous malignancies, overexpression of cyclin D1 alone is insufficient to drive transformation. In contrast, cyclin D1 mutants refractory to phosphorylation-dependent nuclear export and degradation are acutely transforming. This raises the question of whether overexpression of cyclin D1 is a significant contributor to tumorigenesis or an effect of neoplastic transformation. Significantly, recent work strongly supports a model wherein nuclear accumulation of cyclin D1-dependent kinase during S-phase is a critical event with regard to transformation. The identification of mutations within SCF^Fbx4-αBcrystallin ^ligase in primary tumors provides mechanistic insight into cyclin D1 accumulation in human cancer. Furthermore, analysis of mouse models expressing cyclin D1 mutants refractory to degradation indicate that nuclear cyclin D1/CDK4 kinase triggers DNA re-replication and genomic instability. Collectively, these new findings provide a mechanism whereby aberrations in post-translational regulation of cyclin D1 establish a cellular environment conducive to mutations that favor neoplastic growth.

## Introduction

Mitogenic signalling induces transcription and translation of the D-type cyclins, the allosteric regulators of CDK4/6, during G1 phase coupling growth stimuli to cell cycle progression [1]. Active cyclin D1/CDK4 complexes translocate to the nucleus and phosphorylate the retinoblastoma protein (Rb) and related pocket proteins, thereby triggering E2F-dependent transcription of genes required for S-phase entry [2-6]. The timely expression and accumulation of cyclin D1 is ensured through several mechanisms. Initially, *cyclin D1 *expression requires activation of the Raf-Mek-Erk kinase cascade [7-10]. This increased expression is accompanied by phosphatidylinositol 3-kinase and Akt-dependent increases in cyclin D1 translation and decreased cyclin D1 protein degradation [11-13]. Following the G1/S transition, cyclin D1 is rapidly phosphorylated by GSK3β on Thr-286, triggering CRM1-dependent nuclear export [13]. Thr-286 phosphorylated cyclin D1 is recognized by Fbx4 and the co-factor αB crystallin and is subsequently poly-ubiquitylated and degraded by the 26S proteasome [14].

While cyclin D1 overexpression occurs frequently in human cancer and is considered a causative factor in many tumor types, simple over-expression of wild type cyclin D1 is insufficient to drive neoplastic transformation [15]. In contrast, cyclin D1 mutants refractory to phosphorylation and subsequent cytoplasmic proteasomal degradation are acutely transforming *in vitro *and *in vivo *[15,16] implying that compartmentalization of cyclin D1 complexes is essential for cell homeostasis. Indeed, mutations that directly impact on cyclin D1 nuclear export and subsequent proteolysis have been identified in human tumors [17,18]. However, the occurrence of such mutations is rare compared to the frequency of cyclin D1 overexpression. Implicit to this data, if cyclin D1 is a driver oncogene, its overexpression in many cancers must be secondary to tumor-specific alterations that modify its subcellular location during the cell cycle. Here, we discuss recent work to address these questions.

### Cancer-derived cyclin D1 mutations

Cyclin D1 overexpression occurs in carcinomas of the breast, esophagus, head and neck, and lung; in a majority of these cases, alterations in gene expression cannot account for its overexpression. Perturbations in cyclin D1 degradation have been suggested as the primary contributor in a large percentage of cases. Indeed mutations that interfere with Thr-286 phosphorylation occur, but are rare. Such mutations have been noted in endometrial and esophageal cancer [17,18]. For example, of single-base substitutions in the *CCND1 *gene changing proline-287 (Pro-287) to a serine or threonine residue in endometrioid endometrial carcinoma correlates with overexpression of cyclin D1 in the nucleus of neoplastic cells. Additionally, a 12-base pair in frame deletion corresponding to deletion of amino acids 289–292 was reported with overexpression of cyclin D1 [17]. Significantly, subsequent analyses revealed that disruption of Pro-287 abrogates GSK3β-dependent phosphorylation of Thr-286, resulting in nuclear localization of cyclin D1, and deletion of residues 289–292 impairs cyclin D1 binding to CRM1, also resulting in nuclear accumulation [18,19].

In accordance with endometrial cancer studies, recently identified cyclin D1 mutations in esophageal cancer and tumor-derived cell lines also disrupt Thr-286 phosphorylation [18]. Sequencing of cyclin D1 (*CCND1*) in a panel of 90 patient esophageal carcinomas revealed mutation of Thr-286 to arginine and a deletion of C-terminal residues 266–295. Additionally, screening of human tumor-derived esophageal carcinoma cell lines also identified a Pro-287 to alanine mutation in three of these lines [18].

Alternative splicing may also contribute to cancer specific accumulation of cyclin D1 proteins that cannot undergo cytoplasmic degradation [20]. Tumor-specific alternative splicing produces a truncated transcript lacking exon 5, the region encoding the C-terminal Thr-286; this cyclin D1 transcript b (D1b) produces a constitutively nuclear protein refractory to proteasomal degradation [21]. Current work suggests that D1b may be expressed in up to 40% of primary esophageal carcinomas [21]. While cyclin D1b stability is only moderately increased relative to wild type protein, it is refractory to nuclear export and therefore exhibits constitutively nuclear localization. As might be anticipated, expression of cyclin D1b promotes neoplastic transformation *in vitro*, much like the phosphorylation-deficient T286A mutant [15,21]. It is currently unclear what determines alterations in cyclin D1 splicing; polymorphisms that occur in the splice-donor site at the exon 4/intron boundary have been implicated. However, cyclin D1b has been detected in cells that do not exhibit polymorphic residues implicating the occurrence of alternative mechanisms [21].

### Disruption of SCF^Fbx4-αBcrystallin ^function: novel insights into cyclin D1 overexpression

Cell cycle progression is driven by alternating phases of cyclin expression and destruction. Degradation is coordinated by substrate ubiquitylation and destruction via the 26S proteasome [22,23]. Poly-ubiquitylation of substrate proteins is catalyzed by the sequential activity of a ubiquitin activating enzyme (E1), ubiquitin conjugating enzyme (E2), and ubiquitin ligase (E3) [24]. The Skp1-Cul1-F box (SCF) E3 ubiquitin ligases facilitate polyubiquitylation of phosphorylated substrates; among the substrates are many of the key regulators of G1 progression [25]. We recently identified the E3 ubiquitin ligase that directs phosphorylation-dependent polyubiquitylation of cyclin D1, SCF^Fbx4-αB crystallin ^[14]. Substrate recognition by this ligase is analogous with Skp2/Cks1 in that it is directed by the F box protein Fbx4 in concert with a cofactor, αB crystallin [14].

Given the low frequency of mutations within cyclin D1 that directly impact is turnover, a logical prediction was the occurrence of inactivating mutations in its E3 ligase. The first clue to cyclin D1 ligase involvement came from analysis of several breast cancer cell lines exhibiting increased cyclin D1 half-life without mutations disrupting phosphorylation. These analyses revealed that MCF-7 and MDA-MB-231 cells lack αB crystallin expression as a consequence of chromosome deletions. In addition, tumor microarray analysis of esophageal carcinomas revealed a reduction in both αB crystallin and Fbx4 mRNA levels in tumor tissues [14]. Collectively, these findings implicated the cyclin D1 ligase as a target in tumorigenesis, with proteins such as αB crystallin or Fbx4 functioning as putative tumor suppressors.

Significantly, recent assessment of the *Fbx4 *sequence in primary esophageal carcinoma samples identified hemizygous, missense mutations in 14 percent of the tumors; no mutations occurred in α*B crystallin *or *CCND1 *genes in tumors expressing mutated Fbx4. A high percentage of mutations reside within a putative Fbx4 dimerization domain, while others target Ser-12 in the N-terminus. Additionally, one mutation identified within the F box domain results in production of a dominant negative protein incapable of recruiting Skp1 and Cul1[26]. The residues targeted in cancer suggest a model wherein Fbx4 is phosphorylated on Ser-12 and functions as a dimer. While substrate phosphorylation serves as a critical step in regulating SCF ligase function, several studies indicated that F box proteins such as Fbw7 and β-TrCP can form dimers, potentially regulating ligase activity [27-30].

Further analysis of the cyclin D1 ligase revealed that GSK3β, the kinase responsible for cyclin D1 Thr-286 phosphorylation, also catalyzes phosphorylation of Fbx4 Ser-12. Fbx4 phosphorylation correlates with low cyclin D1 expression during G2/M and early G1, with a marked decrease during cell cycle entry due to growth factor-dependent GSK3β inhibition [11]. Fbx4 phosphorylation increases again at the G1/S boundary as GSK3β becomes active and temporally controls ligase activation and cyclin D1 phosphorylation. These findings raise the question of how Fbx4 phosphorylation regulates ligase function. Additional work demonstrated that Fbx4 forms homodimers in a phosphorylation- and cell cycle-dependent manner [26]. Fbx4 homodimerization is dependent on Ser-12 phosphorylation; disruption of this residue impairs the ubiquitylation activity of this ligase [26].

The tumorigenic potential of Fbx4 mutations disrupting phosphorylation and dimerization was assessed *in vitro*, revealing that such mutations are indeed transforming [26]. These findings suggest that mutation of Ser-12 or residues within the Fbx4 dimerization domain impairs ligase function, contributing to cyclin D1 accumulation. Studies over the past several years have identified mutations in both cyclin D1 and its E3 ubiquitin ligase that impair proteasomal degradation and promote nuclear accumulation of cyclin D1/CDK4 complexes (summarized in Table 1). Therefore, delineating the mechanism underlying nuclear cyclin D1-driven transformation is critical for understanding the role of cyclin D1 in tumorigenesis and development of therapeutic strategies for treatment of cancers overexpressing this protein.

**Table 1:** Summary of mutations targeting cyclin D1 phosphorylation or ligase function

Target	Mutation	Consequence	Tumor Type	Reference
Cyclin D1	T286R	Constitutively Nuclear	Esophageal	[18]
Cyclin D1	Δ266–295	Constitutively Nuclear	Esophageal	[18]
Cyclin D1	P287A	Constitutively Nuclear	Tumor-derived esophageal carcinoma cell lines TE3, TE7, and TE12	[18]
Cyclin D1	P287S/T	Constitutively Nuclear	Endometrial	[17]
Cyclin D1	Δ289–292	Constitutively Nuclear	Endometrial	[17]
αB crystallin	Chromosome 11 deletion	Impaired ligase activity	Tumor-derived breast cancer cell lines (MCF-7, MDA-MB 231)	[14]
Fbx4	S8R	Impaired ligase activity	Esophageal	[26]
Fbx4	S12L	Disrupts phosphorylation	Esophageal	[26]
Fbx4	P13S	Disrupts phosphorylation	Esophageal	[26]
Fbx4	L23Q	Dimerization-deficient	Esophageal	[26]
Fbx4	P76T	Impaired Skp1 binding	Esophageal	[26]

Mutations disrupting GSK3β-dependent cyclin D1 phosphorylation and nuclear export include mutation of Thr-286, Pro-287, and deletion of residues corresponding to the CRM1 binding site. Mutations targeting the SCF^Fbx4-αB crystallin ^E3 ubiquitin ligase result in impaired ligase activity and subsequent cyclin D1/CDK4 accumulation in the nucleus.

### Inhibition of cyclin D1 degradation promotes genomic instability

Aberrant nuclear accumulation of cyclin D1 during S-phase drives cell transformation *in vitro *and B-cell lymphomagenesis in mice [15,16]. Recent evidence links nuclear retention of active cyclin D1/CDK4 complexes with genomic instability, providing a novel mechanism wherein cyclin D1 stabilization initiates tumor formation. Nuclear accumulation of active cyclin D1-dependent kinase was found to interfere with Cdt1 proteolysis [31]. Cdt1 is a component of the pre-replicative complex that promotes loading of the replicative helicase during late G1 phase [32,33]. The failure to degrade Cdt1 during S-phase has been shown to contribute to DNA re-replication in several systems [34,35]. In cells harbouring nuclear cyclin D1, Cdt1 proteolysis was disrupted due to repression of Cul4A and Cul4B expression. Cul4 proteins serve as scaffolds for the E3 ligase that regulates Cdt1 [36,37]. Ultimately, nuclear cyclin D1/CDK4 complexes facilitate stabilization of Cdt1 during S-phase, with concurrent maintenance of the MCM helicase on chromatin, resulting in DNA re-replication and activation of DNA damage checkpoint signalling [31].

**Figure 1:**
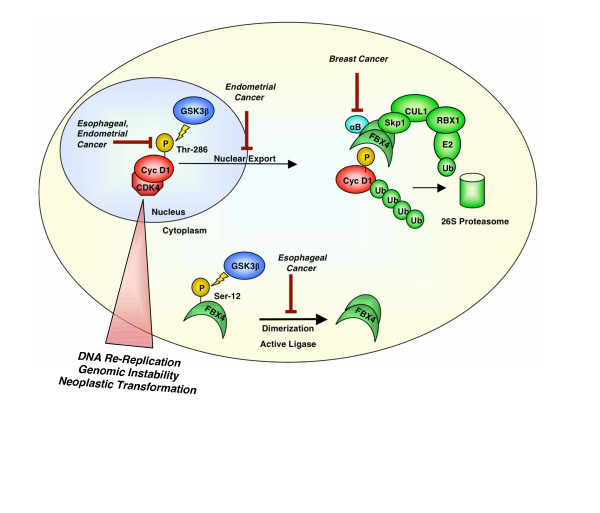
**Cyclin D1 Regulatory Pathways are Targeted in Human Cancer**. Cyclin D1 protein accumulation is tightly controlled via phosphorylation-dependent proteolysis. Mutations targeting cyclin D1 phosphorylation or degradation contribute to neoplastic transformation. Specific disruption of Thr-286 phosphorylation occurs in endometrial and esophageal carcinoma, while mutations preventing Crm1 binding occur in endometrial cancer. Mutations targeting Fbx4 have been identified in esophageal cancer, and αB crystallin loss occurs in tumor-derived breast carcioma cell lines. Disruption of cyclin D1 proteolysis promotes accumulation of active cyclin D1/CDK4 kinase, triggering DNA re-replication and subsequent genomic instability necessary to drive neoplastic transformation.

Active cyclin D1/CDK4 complexes influence transcriptional regulation of the Cul4 proteins; however, the precise mechanism of regulation remains to be elucidated. Strikingly, impaired cyclin D1 ligase function results in nuclear accumulation of active cyclin D1/CDK4 complexes, accumulation of Cdt1, and triggers cellular transformation analogous to cyclin D1 T286A [26]. Taken together, data elucidating the role of nuclear cyclin D1 in neoplastic transformation support a model wherein disruption of cyclin D1 phosphorylation or SCF^Fbx4-αBcrystallin ^activation generate genomic instability, ultimately driving tumor formation (Figure 1).

Genomic integrity is monitored by cell cycle checkpoints that promote cell cycle arrest or apoptosis upon detection of damaged DNA [38]. Chronic activation of checkpoints may provide selective pressure for deletion or mutation of critical tumor suppressors such as p53 [39,40]. Consistent with this notion, cyclin D1T286A-dependent tumorigenesis is accompanied by activation of the DNA damage checkpoint pathway and loss of p53 [31]. The deleterious effects of nuclear cyclin D1/CDK4 complexes could then have two different effects on cellular transformation. Chronic checkpoint activation can induce selective pressure for loss of tumor suppressors, thereby providing such cells not only with a growth advantage but also the propensity for additional genomic instability.

### Cyclin D1 protein accumulation in tumors: potential therapeutic strategies

Provided with this new data suggesting that cyclin D1-dependent kinase contributes to neoplasia at least in part through perturbations in DNA replication and loss of genomic integrity, can we utilize this information for increased therapeutic modalities? One scenario might be to take advantage of the fact that tumors harbouring mutations in cyclin D1 or Fbx4 have a compromised DNA damage checkpoint due to loss of p53. In theory, treatment of normal cells with chemotherapeutic agents that generate DNA crosslinks should trigger an intra S-phase checkpoint, thereby providing an opportunity for repair. Expression of stabilized cyclin D1 should promote maintenance of Cdt1 and MCM complexes and in so doing promote continued origin firing without allowing for repair of damaged DNA, ultimately resulting in mitotic catastrophe. Further work is required to investigate how alterations in cyclin D1 proteolysis might influence cellular responses to DNA damaging therapeutics.

The alternative is the development of drugs that directly target the kinase subunits that cyclin D1 regulates. If continued activation of CDK4/6 is required for tumor proliferation and survival, such drugs may have significant clinical use. Consistent with this notion of oncogene addiction, treatment of mammary epithelial cells derived from murine tumors harbouring a MMTV-T286A transgene with the CDK4/6 inhibitor PD0332991 [41] triggered G1 arrest [42]. Therefore, CDK4/6 activity is a potential target to prevent cyclin D1-driven proliferation.

### Concluding Remarks

Phosphorylation-dependent nuclear export and subsequent degradation of cyclin D1 is essential to maintain cellular homeostasis. Disruption of this regulatory pathway has been extensively shown to promote neoplastic transformation; however, the precise mechanism of this event has been elusive. Significantly, recent work revealed that nuclear accumulation of active cyclin D1/CDK4 complexes generates genomic instability through a mechanism of Cdt1 stabilization and DNA re-replication. Furthermore, mutations targeting SCF^Fbx4-αB crystallin ^in human cancers implicate ligase function in cyclin D1 overexpression and subsequent nuclear accumulation of active cyclin D1/CDK4 complexes. Importantly, GSK3β functions as the master switch, turning on cyclin D1 destruction at the G1/S transition by regulating both ligase activation and cyclin D1 phosphorylation. Given the phenotypic outcome of accumulated nuclear cyclin D1/CDK4 complexes, further mechanistic investigation is required for development of novel therapeutic strategies to promote tumor cell death in cancers overexpressing cyclin D1.

## Competing interests

The authors declare that they have no competing interests.

## Authors' contributions

LLP and JAD contributed to the discussion and preparation of this manuscript.
